# Bevacizumab Demonstrates Prolonged Disease Stabilization in Patients with Heavily Pretreated Metastatic Renal Cell Carcinoma: A Case Series and Review of the Literature

**DOI:** 10.1155/2010/687043

**Published:** 2010-07-20

**Authors:** Nicole M. Agostino, Rebecca Gingrich, Joseph J. Drabick

**Affiliations:** Department of Internal Medicine, Division of Hematology and Oncology, Penn State Milton S. Hershey Medical Center, Hershey, PA 17078, USA

## Abstract

There are now a variety of therapies approved for the treatment of metastatic renal cell carcinoma (RCC). These include the immunotherapeutics, alfa-interferon, and interleukin-2, and agents that target the vascular endothelial growth factor receptor (VEGFR) via its tyrosine kinase, such as sorafenib, sunitinib, and pazopanib, or the mammalian target of rapamycin (mTOR), such as temsirolimus and everolimus. Bevacizumab, a monoclonal antibody directed against the ligand, VEGF, has shown activity against RCC as a single agent in patients who had failed prior cytokine therapy and as first line therapy in combination with interferon. The activity of bevacizumab in patients who had received and failed prior therapy has not been described. We report our experience in 4 patients with metastatic RCC who had failed prior cytokine, TKI, and mTOR inhibitors who were treated with bevacizumab as single agent therapy. These heavily pretreated patients sustained very prolonged periods of stable disease (median of 12 months) with very little toxicity and excellent quality of life. The activity of this agent in patients who had failed prior therapies directed against the VEGFR and mTOR suggests that therapy targeting the ligand, VEGF, is still a viable approach in these patients and deserves further study.

## 1. Introduction

Last year in the United States an estimated 54,390 Americans were diagnosed with renal cell carcinoma (RCC) and 13,010 died from the disease. The rate of RCC has also been increasing by 2% per year for the past 65 years with smoking, obesity, hypertension, cystic kidney disease, and mutation of the von-Hippel Lindau tumor suppressor gene being significant risk factors [1, 2]. Unfortunately, 30% of patients present with metastatic disease [3].

Renal cell carcinoma does not response to traditional chemotherapeutic agents and until the earlier part of this decade, biologic response modifiers such as interleukin-2 (IL-2) and interferon-*α* (IFN-*α*) were the only available treatments [1, 4]. More recently, with an improved understanding of the biology of RCC, targeted therapies such as sorafenib, sunitinib, pazopanib, temsirolimus, and everolimus have proven to be effective therapies in the metastatic setting [1]. These agents target the tyrosine kinase of the VEGFR or the mTOR kinase in the PIK3CA/Akt signaling pathway. Bevacizumab, a recombinant monoclonal antibody against the vascular endothelial growth factor-A (VEGF-A) has also been used as first line therapy in Phase III trials in combination with IFN-*α* with good results [5, 6] leading to its approval in metastatic RCC. The activity of bevacizumab in patients who had already tried and failed TKIs and/or mTOR inhibitors has not been described. We present a case series of four patients to illustrate our institutional experience with single agent bevacizumab as an option for patients who have failed all other available therapies.

## 2. Case Reports

### 2.1. Case One

Patient A was a 73-year-old white male who was diagnosed with RCC in 2000 and subsequently underwent a left radical nephrectomy. He went on to have multiple locoregional recurrences in the nephrectomy bed and in the lower pole of the right kidney. By January of 2007, he had involvement of the pancreatic tail and the left adrenal gland as well. He was evaluated at our medical center in March of 2007. He was restaged at that point and it was confirmed that he had no disease outside of the abdomen, so he was offered radical resection of his disease with curative intent by surgical oncology, but the patient declined. Therefore, he was started on sunitinib 50 mg daily for 4 weeks of a 6 week cycle, and an MRI in July 2007 showed decrease in the size of the tumor in the left renal bed and the pancreatic mass and right renal pole mass remained stable. Imaging after 24 weeks of sunitinib showed stable disease in known sites. His disease remained stable until imaging performed in April of 2008, after 36 weeks of treatment, showed clear enlargement of the renal fossa mass. The patient was then started on temsirolimus 25 mg IV weekly for second-line therapy. The patient had thrombocytopenia, worsening of lipid profile, and elevated blood glucose levels on temsirolimus that required a 20% dose reduction. After 12 weeks, CT scan showed pneumonitis consistent with mTOR inhibitor induced pneumonitis, a possible new lung metastasis, and progression of disease in the liver and renal fossa. Single agent bevacizumab 10 mg/m^2^ given every 2 weeks was started for third-line therapy and restaging every 3 months showed continued stable disease. The patient enjoyed 12 months of stable disease on single agent bevacizumab. It was stopped despite stable disease due to development of osteonecrosis of the jaw and patient request. The patient subsequently progressed rapidly and expired while off therapy.

### 2.2. Case Two

Patient B is a 76-year-old white male who presented with microscopic hematuria in 1986 on a routine urinalysis and a diagnosis of left RCC was made by imaging. He then underwent nephrectomy for a 7 × 6 × 5.5 cm tumor extending though the fascia into the perinephric fat. He did well until March of 2006 when he had a routine chest X-ray that showed new lung nodules and a CT scan was ordered. A large retroperitoneal mass posterior to the stomach measuring 10 × 9 × 8 cm was also found along with bilateral lung nodules, with the largest lung lesion measuring 2 cm. Biopsy of the retroperitoneal mass confirmed RCC. MRI of the brain and bone scan was negative. He was seen for the first time at our institution in April of 2006. He was determined to be a candidate for high-dose IL-2 (600,000 IU/kg). After 8 weeks, he did show some response, but was only able to tolerate half of cycle 2 and he declined further IL-2. He was then started on sorafenib and had a minor response at all disease sites until CT scan in January 2007 after 24 weeks showed progression. He was then switched to sunitinib 50 mg daily for 4 weeks of a 6 weeks cycles, and after 12 weeks on therapy had a significant response which he enjoyed until week 54 when CT scanning showed marked disease progression. Sunitinib was discontinued and in January of 2008 he was started on temsirolimus 25 mg IV weekly. He required a 20% dose reduction for thrombocytopenia and oral ulcers. He remained on temsirolimus until April of 2008 when he developed symptomatic mTOR interstitial pneumonitis, so temsirolimus was discontinued. His disease continued to progress off therapy and in October of 2008, bevacizumab 10 mg/m^2^ given every 2 weeks was started as fifth line therapy. After 24 weeks of treatment the patient was found to have a pulmonary embolus. Lovenox was started and bevacizumab was continued without incident. At the time of submission, the patient's disease has remained stable after 72 weeks of bevacizumab. He has continued to work full time as a maintenance man.

### 2.3. Case Three

Patient C is a 68-year-old white man who was diagnosed with metastatic RCC, clear cell type in July 2008 discovered as part of an evaluation for new onset atrial fibrillation. He had a 7.1 × 9.7 cm left renal mass, enlarged retroperitoneal and mediastinal lymph nodes and multiple lung nodules. Mediastinal lymph node was positive for metastatic RCC on biopsy. He underwent debulking nephrectomy in August 2008 and then had a trial of high-dose IL-2 (600,000 IU/kg) which failed with progressive disease. He began sunitinib 50 mg daily for 4 weeks of a 6 week cycle, and had initially stable disease, but progressed after 36 weeks. He then began everolimus 10 mg daily but developed symptomatic mTOR-inhibitor-associated pneumonitis which resolved with steroids and discontinuation of everolimus. He then began bevacizumab 10 mg/m^2^ given every 2 weeks and had an initial minor response then prolonged stable disease. He remains on therapy at 24 weeks to date. He continues to be very active, golfing regularly and traveling.

### 2.4. Case Four

Patient D is a 58-year-old white female who was diagnosed with a T2, N0, M0 right sided RCC of clear cell histology in 2001. She underwent radical nephrectomy and was followed by her urologist who found a small enlarging left adrenal mass and numerous pulmonary nodules concerning for metastatic disease in 2005. Metastatic disease was confirmed via biopsy of a lung lesion and the patient was started on high-dose IL-2 (600,000 IU/kg). After 8 weeks, she had disease progression on CT scan in both the lung and adrenal gland. She was then treated with sunitinib 50 mg daily for 4 weeks of a 6 week cycle, in the context of a clinical trial. She required a dose reduction because of pancytopenia. Imaging after 30 weeks showed stable pulmonary disease and improvement of the adrenal lesion and retroperitoneal lymphadenopathy. She remained on sunitinib with stable disease until September 2007 when CT scan showed disease progression in aortocaval lymph nodes. Her treatment was then changed to sorafenib 400 mg BID. She achieved 54 weeks of stable disease until July 2008 when her disease progressed on CT scan. The patient was then put on temsirolimus 25 mg IV weekly until October of 2008 when, once again, she had disease progression on CT scan. She was placed on bevacizumab 10 mg/m^2^ every 2 weeks and completed 48 weeks of treatment. She then progressed on bevacizumab at all sites and began 6th-line treatment with pazopanib 800 mg daily. She continues to be active and well.

## 3. Discussion

Until recently, treatment options metastatic RCC were limited to cytokine therapy with IFN-*α* and/or high-dose IL-2 [1]. Sunitinib, pazopanib, and sorafenib are multi-TKIs that between them affect VEGF, c-kit, platelet derived growth factor-beta (PDGFR-*β*), FLT3, and BRAF [7, 8]. These drugs have been approved for metastatic RCC as first line or second line [9–11]. The mTOR inhibitors, temsirolimus and everolimus, are recommended for poor prognosis patients as first line and for second line after TKI failure, respectively. The mTOR (mammalian Target of Rapamycin) regulates many downstream signally paths, including the HIF-1, and controls metabolism, cell growth and angiogenesis [12, 13]. 

We report our experience with the recombinant monoclonal antibody to vascular endothelial growth factor A (VEGF-A), bevacizumab, in heavily pre-treated patients with RCC. This agent was originally evaluated in a phase II trial of 116 patients with metastatic RCC who had failed high-dose IL-2. Patients were randomized to receive low-dose bevacizumab (3 mg/kg), high-dose bevacizumab (10 mg/kg), or placebo. Time to progression (TTP) and response rate (RR) were evaluated as primary endpoints. In the high-dose group only, there was a significant increase in TTP compared to placebo (4.8 months versus 2.5 months, *P* < .001). The response rate of high-dose bevacizumab was 10% in this study, with one patient having a partial response (PR) for the maximum treatment period of two years. This patient stopped therapy after the trial and subsequently had a relapse six months later, but enjoyed a second PR after restarting bevacizumab under a compassionate exemption [14].

The AVOREN trial, a multicenter, randomized, double blind phase III trial of 641 patients were treated in the first-line setting with bevacizumab (10 mg/kg every 2 weeks) plus IFN-*α* (9 MIU 3 times weekly) or placebo plus IFN-*α*. Patients on the bevacizumab arm experienced a progression free survival (PFS) of 10.2 month versus 5.4 months in the placebo arm. Response rate was 30.6% in the bevacizumab arm versus 12.4% in the placebo arm. There was also a trend towards improved overall survival [5]. A similar study in the United States, CALGB 90206, randomized patient to receive first-line bevacizumab (10 mg/kg every 2 weeks) plus IFN-*α* (9 MIU 3 times weekly) or IFN-*α* alone. Once again, the bevacizumab arm produced a superior PFS of 8.5 months compared to 5.2 months in the IFN-*α* only arm. Response rate was 25.5% in the bevacizumab arm versus 13.1% in the IFN-*α* alone arm [6]. Patients in both of these studies had not received any prior systemic treatment for RCC.

The activity of bevacizumab as a single agent in patients who had already tried and progressed on TKIs and mTOR inhibitors has not been previously described. Pastorelli and colleagues recently published a single case report of a 67-year-old female with metastatic RCC that was treated in the second line setting with IFN-*α* (3 MIU three times weekly) and bevacizumab (10 mg/kg) together after achieving a brief PR on sunitinib. Unfortunately, she had significant toxicities from sunitinib that required dose reductions and prevented her from switching to another TKI after progression. She then achieved a PR for eight months on IFN-*α* and bevacizumab. Despite the fact that TKIs and bevacizumab both have antiangiogenic properties, cross-resistance did not occur in this case and supports our observations [15]. 

Table 1 lists the characteristics of our 4 responding patients. To date, we have treated 6 patients in our practice with late-line bevacizumab after failure of all available prior therapies. Two patients not presented tolerated treatment well but did not respond and had clear disease progression on assessment after 2 to 3 cycles. The other 4 patients however, exhibited prolonged disease stabilization (median 12 months) with minimal toxicity and excellent quality of life. The response rate in the 6 patients treated in our practice was 67%. In the palliative setting, the side effect profile of bevacizumab is an important consideration. The most significant and consistent adverse events from clinical trials of bevacizumab were proteinuria, hypertension, fatigue, asthenia, and cytopenias. Bleeding and venous thromboembolism are always a serious concern when giving anti-VEGF therapy [14]. In our short case series, Patient B did experience a pulmonary embolus while on bevacizumab, but this drug was otherwise well tolerated and he continued therapy while on anticoagulation. Patient A developed osteonecrosis of the jaw, an unusual but reported side effect of prolonged bevacizumab [16]. At the time of this report, three of the four patients whom we have treated had enjoyed over a year of stable disease on single agent bevacizumab with an acceptable side effect profile and good quality of life. 

Bevacizumab retained a significant prolonged disease stabilization effect after prior failure of both TKIs and mTOR inhibitors, an observation which has important biological implications. This suggests that the signaling pathways within the cytoplasm may be altered to confer resistance to agents that are active in the cytoplasm such as the TKIs and mTOR inhibitors, but the pathway is still intact so that interference with the ligand, VEGF, binding to its receptor may still suppress tumor growth. Our clinical experience suggests that bevacizumab should be studied in a larger group of heavily pre-treated patients in the context of a clinical trial to better define its activity. The high-response rate and tolerability noted in these heavily pre-treated patients in real clinical practice is certainly encouraging.

## Figures and Tables

**Table 1:** Patient characteristics and responses to treatments.

Patient ID	Age	Gender	Prior Treatment	Responses to Prior Treatment	Bevacizumab best response and duration of response
A	73	M	Sunitinib	SD for 54 weeks	SD for 48 weeks
			Temsirolimus	PD after 3 cycles (3 months)	

B	76	M	IL-2	PR after 12 weeks	SD for 72 weeks*
			Sorafenib	PR for 24 weeks	
			Sunitinib	PR for 54 weeks	
			Temsirolimus	AE after 12 weeks	

C	56	M	IL-2	PD after 8 weeks	SD for >24 weeks*
			Sunitinib	SD for 24 weeks	
			Temsirolimus	AE after 12 weeks	

D	58	F	IL-2	PD after 12 weeks	SD for 48 weeks
			Sunitinib	SD for 84 weeks	
			Sorafenib	SD for 36 weeks	
			Temsirolimus	PD after 12 weeks	

SD: Stable disease, PD: Progression of disease, PR: Partial response, AE: Adverse event.

*Patient still on therapy at the time of publication.

## References

[1] National Comprehensive Cancer Network Kidney Cancer. http://www.nccn.org/.

[2] Cohen HT, McGovern FJ (2005). Renal-cell carcinoma. *New England Journal of Medicine*.

[3] DeVita VT, Hellman S, Rosenberg SA (2008). *Cancer Principle and Practice of Oncology*.

[4] Yagoda A, Abi-Rached B, Petrylak D (1995). Chemotherapy for advanced renal-cell carcinoma: 1983–1993. *Seminars in Oncology*.

[5] Escudier B, Pluzanska A, Koralewski P (2007). Bevacizumab plus interferon alfa-2a for treatment of metastatic renal cell carcinoma: a randomised, double-blind phase III trial. *The Lancet*.

[6] Rini BI, Halabi S, Rosenberg JE (2008). Bevacizumab plus interferon alfa compared with interferon alfa monotherapy in patients with metastatic renal cell carcinoma: CALGB 90206. *Journal of Clinical Oncology*.

[7] Oxelmark E, Hornberg JJ (2007). Finding the way in the jungle of kinase drug targets. *Drug Discovery Today: Technologies*.

[8] Wilhelm S, Carter C, Lynch M (2006). Discovery and development of sorafenib: a multikinase inhibitor for treating cancer. *Nature Reviews Drug Discovery*.

[9] Motzer RJ, Hutson TE, Tomczak P (2007). Sunitinib versus interferon alfa in metastatic renal-cell carcinoma. *New England Journal of Medicine*.

[10] Figlin RA, Hutson TE, Tomczak MD (2008). Overall survival with sunitinib versus interferon (IFN)-alfa as first-line treatment of metastatic renal cell carcinoma (mRCC). *Journal of Clinical Oncology*.

[11] Szczylik T, Demkow M, Staehler F (2007). Randomized phase II trial of first-line treatment with sorafenib versus interferon in patients with advanced renal cell carcinoma: final results. *Journal of Clinical Oncology*.

[12] Hudes G, Carducci M, Tomczak P (2007). Temsirolimus, interferon alfa, or both for advanced renal-cell carcinoma. *New England Journal of Medicine*.

[13] Motzer RJ, Escudier B, Oudard S (2008). Efficacy of everolimus in advanced renal cell carcinoma: a double-blind, randomised, placebo-controlled phase III trial. *The Lancet*.

[14] Yang JC, Haworth L, Sherry RM (2003). A randomized trial of bevacizumab, an anti-vascular endothelial growth factor antibody, for metastatic renal cancer. *New England Journal of Medicine*.

[15] Pastorelli D, Zustovich F, Faggioni G (2010). Good response to second-line bevacizumab and interferon-*α* in a sunitinib-refractory patient with metastatic renal cell carcinoma. *Anti-Cancer Drugs*.

[16] Estilo CL, Fornier M, Farooki A, Carlson D, Bohle G, Huryn JM (2008). Osteonecrosis of the jaw related to bevacizumab. *Journal of Clinical Oncology*.

